# Dietary Corn Bran Altered the Diversity of Microbial Communities and Cytokine Production in Weaned Pigs

**DOI:** 10.3389/fmicb.2018.02090

**Published:** 2018-09-04

**Authors:** Ping Liu, Jinbiao Zhao, Wei Wang, Pingting Guo, Wenqing Lu, Chunlin Wang, Ling Liu, Lee J. Johnston, Yuan Zhao, Xianhua Wu, Chi Xu, Jie Zhang, Xi Ma

**Affiliations:** ^1^State Key Laboratory of Animal Nutrition, College of Animal Science and Technology, China Agricultural University, Beijing, China; ^2^Swine Nutrition and Production, West Central Research and Outreach Center, University of Minnesota, Morris, MN, United States; ^3^Ningxia DaBeiNong Science and Technology Co., Ltd. (DBN), Yinchuan, China; ^4^Department of Animal Husbandry and Veterinary, Beijing Vocational College of Agriculture, Beijing, China; ^5^Department of Internal Medicine, Department of Biochemistry, University of Texas Southwestern Medical Center, Dallas, TX, United States; ^6^College of Animal Science and Technology, Qingdao Agricultural University, Qingdao, China

**Keywords:** corn bran, fermentation, fibrolytic bacteria, inflammation, short-chain fatty acids, weaned pigs

## Abstract

Corn bran (CB) has been used as an ingredient for pigs, but the underlying mechanisms that improve gut health is less clear. This study was conducted to investigate effects of dietary CB on growth performance, nutrient digestibility, plasma indices related to gut hormones and immunity, gut microbiota composition, and fermentation products in weaned pigs. A total of 60 weaned pigs were allocated to two dietary treatments, and piglets in each group received control (CON) diet or 5% CB diet for 28 days. Growth performance, nutrient digestibility, indices of gut hormones and immunity in plasma were evaluated. Microbiota composition in feces was determined using 16S rRNA amplicon sequencing, and fermentation products were measured by high-performance ion chromatography. The results showed that dietary CB did not affect growth performance, nutrient digestibility, gut hormones, or fermentation products in the trial (*P* > 0.05). There was an increased response to CB inclusion on interleukin-10 production (*P* < 0.05). On day 28, piglets fed dietary CB had a higher shannon index (*P* < 0.05). The population of the *Firmicutes* in CB treatment were decreased (*P* < 0.05), while the percentage of the *Bacteroidetes* were increased (*P* < 0.05). In particular, the populations of *Eubacterium corprostanoligenes, Pevotella*, and *Fibrobacter* related to polysaccharide fermentation of cereal bran were increased (*P* < 0.05). In conclusion, a post-weaning diet containing 5% CB increased intestinal microbial diversity, especially higher richness of fibrolytic bacteria, and promoted anti-inflammatory response to some extent in piglets, these changes should facilitate the adaptation of the digestive system of piglets in the subsequent growing phases.

## Introduction

Dietary fiber (DF) plays important roles in improving immune defense and maintaining microbial ecosystem (Martens, 2016; Daien et al., 2017). It has been emphasized in the discipline of animal nutrition, DF can be applied as prebiotic to improve animal health and reduce the usage of antimicrobials in animal husbandry (Slavin, 2013). There are numerous fiber sources from plants and the effects of DF largely depend on their physicochemical properties, such as fermentability, water solubility, and glycan structures (Molist et al., 2014). Main sources of soluble and readily fermentable fiber are from vegetables and fruits, while cereal fibers are mostly insoluble and less fermentable (Weickert et al., 2006). DF is known to be resistant to enzymatic hydrolysis by endogenous digestive enzymes and can be utilized as a carbon source for the growth of bacterial community through the process of fermentation. The end-products of DF are short-chain fatty acids (SCFAs), mainly acetate, propionate, and butyrate, which provide energy substrates for colonocytes, modulate satiety, and alleviate inflammation (Koh et al., 2016). SCFAs can bind to the G protein-coupled receptors (GPRs) that are expressed on both intestinal epithelium and immune cells (Daien et al., 2017). For example, these bacterial metabolites stimulate the release of the anorectic gut hormones such as glucagon-like peptide-1 and peptide YY via activation of GPR43 on enteroendocrine L-cells to exploit the important role in modulating appetite and gut motility (Chambers et al., 2015). Previous studies have shown that high fiber in diets can increase postprandial satiety and decrease subsequence hunger, which resulting in less energy intake and weight loss (Chambers et al., 2015; Zhao L. et al., 2018). In animal studies, the observed effects of DF on animal growth are not consistent due to diverse microbiota-accessible carbohydrates used in different studies. Soluble fiber, such as sugar-beet pulp, can reduce feed intake due to increases of mucus viscosity and digesta transit time (Montagne et al., 2012). Insoluble fiber in diet can increase feed intake of pigs that should be associated with low availability energy in diets and reduction of intestinal transit time (Gerritsen et al., 2012). Moreover, high cereal fiber may lead to reductions of nutrient digestibility (Chen et al., 2013). Therefore, animal growth and macronutrient absorption should be highly considered when diet intervention was carried out in domestic animals.

On the other hand, insoluble fiber in diet can increase fermentation in the hindgut that may alter bacterial community composition and immune response. For instance, wheat bran could induce prebiotic effects in the microbial ecosystem and enhance intestinal epithelial barrier functions in weaning pigs (Gerritsen et al., 2012; D’hoe et al., 2018). The bioactive components of wheat bran, especially arabinoxylan, increased intestinal butyrate production and higher population of *Bifidobacterium* in the large intestine (Chen et al., 2014, Chen H. et al., 2015). SCFAs derived from DF play as modulators on immune cells. Previous studies showed reductions on inflammatory markers (Jiminez et al., 2016) and increased antibody production (Kim et al., 2016) when subjects was supplied high fiber in diets.

Corn bran (CB) is co-products of food industry and has been widely applied in animal feed due to its economic price and wide availability. It is rich in insoluble fiber and mainly composed of arabinoxylan (Damen et al., 2011). However, the association between CB, gut microbiota, and immune response in weaned pigs is less clear. It was hypothesized that long-term intervention of dietary CB might promote the establishment of robust and stable microbiota and enhance immune defense in post-weaning pigs. Therefore, the objective of this study was to investigate the effects of 5% CB inclusion on growth performance, nutrient digestibility, indices related to gut hormones and immunity in blood plasma, gut microbiota composition, and fermentation products in weaned pigs.

## Materials and Methods

### Ethics

This protocol involving pig handling and treatments was carried out in accordance with the recommendations of Laboratory Animals-Guideline of Welfare and Ethics of China. The study was approved by the “Institutional Animal Care and Use Committee of China Agricultural University” (ICS 65.020.30).

### Animals, Diet Treatments, and Sampling

A total of 60 piglets {Duroc × (Landrance × Yorkshire)} were weaned at 26 ± 1 days of age (mean body weight: 7.5 ± 1.2 kg) and randomly allocated to two treatment groups balancing for litter and gender. Piglets were housed in commercial flat-deck pens (five piglets per pen) and *ad libitum* access to feed and water. Room temperature was maintained at 26°C on the day of weaning and gradually decreased to 22°C within the first week after weaning. The humidity was kept constant at 65–75%. Thirty piglets in each group received one of two experimental diets (**Supplementary Table 1**) based on corn and soybean meal, that were the control (CON) diet and 5% CB diet. The treatment lasted for 28 days. No antibiotics were used before and during the trial.

Piglets were weighed individually on days 0, 14, and 28, and feed intake per pen was measured in the experiment. Average daily gain (ADG) and average daily feed intake (ADFI) were calculated. Feed conversion ratio (FCR) was the ratio of ADFI and ADG. Fecal samples (300 g) from each pen (*n* = 6 pens per treatment) were collected on three consecutive days (25–27) in the feeding trial and mixed before drying. Experimental diets and fecal samples were dried in oven at 65°C for 72 h. All samples were smashed to pass through a 1.0-mm mesh screen for the analysis of nutrient digestibility. On days 14 and 28, fresh fecal sample from pigs (*n* = 6 per treatment) were collected from rectal anal junction directly and immediately snap-frozen using liquid nitrogen, and stored at -80°C for the determination of bacterial community composition and fermentation products. Aliquots (5 mL) of blood was withdrawn from the jugular vein of piglets by vacuum blood tube with sodium heparin as an anticoagulant, and samples were centrifuged at 3,000 × *g* for 10 min. The supernatant was collected and stored at -20°C for the measurement of plasma indices related to gut hormones and immunity.

### Fiber Component Analysis

The feed ingredient of CB was purchased from Wellhope Agri-tech Co. Ltd. (Beijing, China). Fiber composition of CB was analyzed, including cellulose, hemicellulose, lignin, insoluble DF (IDF), soluble DF (SDF), total DF (TDF), total non-starch polysaccharides (NSP), and eight constituent monosaccharides of NSP, namely rhamnose, fructose, ribose, arabinose, xylose, mannose, galactose, and glucose. The contents of IDF and TDF were measured according to AOAC method of 991.43 using Ankom Dietary Fiber Analyzer (Ankom Technology, United States). The contents of NSP and their monosaccharides were analyzed on the basis of alditol acetates by gas–liquid chromatography (Agilent GC 6980, United States) as described by Liu et al. (2017). The fiber composition of CB was shown in **Supplementary Table 2**.

### Apparent Total Tract Digestibility of Nutrients

The nutrient content of the experimental diets and feces were analyzed, including gross energy (GE), dry matter (DM), organic matter (OM), crude protein (CP), ether extract (EE), and TDF. Acid insoluble ash (AIA) in diets and feces was analyzed according to the AOAC procedure (AOAC, 2007), and the apparent total tract digestibility (ATTD) of GE, DM, OM, CP, EE, and TDF were calculated using the formula: ATTD (%) = {1 - (AIA_feed_ × Nutrient_feces_)/(AIA_feces_ × Nutrient_feed_)} × 100, in which AIA_feed_ was the AIA concentration in the experimental feed, Nutrient_feces_ was the nutrient concentration in feces, AIA_fece_ was the AIA concentration in feces, and Nutrient_feed_ was the nutrient concentration in the experimental feed.

### Gut Hormones and Immune Indices in Blood Plasma

The concentrations of gut hormones including glucagon-like peptide 1 (GLP1), peptide YY (PYY), and 5-hydroxytryptamine (5-HT) in blood plasma were measured. Moreover, the production of immune indices containing IgA, IgG, IgM, tumor necrosis factor α (TNF-α), interleukin 1β (IL-1β), IL-6, and IL-10 were detected. The levels of IgA, IgG, and IgM were detected by spectrophotometer, and other traits were performed by ELISA assays according to the standard protocols described by manufacturer (Jiancheng Bioengineering Institute, China). The OD value was determined by a microplate reader at 450 nm.

### Extraction of Nucleic Acids and Illumina Sequencing

DNA from feces (*n* = 6 per treatment) was isolated using the E.Z.N.A.^®^ stool DNA Kit (Omega Bio-tek, Norcross, GA, United States) according to manufacturer’s protocols. Final DNA concentration and purification were determined by NanoDrop 2000 UV-vis spectrophotometer (Thermo Scientific, Wilmington, United States), and DNA quality was checked by 1% agarose gel electrophoresis. The V3-V4 regions of the bacterial 16S rRNA gene were amplified using primers F338 (5′-ACTCCTACGGGAGGCAGCAG-3′) and R806 (5′-GGACTACHVGGGTWTCTAAT-3′) with a few modifications to the PCR assay (initial denaturation at 95°C for 3 min, followed by 27 cycles with denaturation at 95°C for 30 s, annealing temperature at 55°C for 30 s, and elongation at 72°C for 30 s, and final extension at 72°C for 10 min). Illumina sequencing was performed as described previously (Liu et al., 2017).

Raw sequences were quality-filtered using Trimmomatic (version 3.29) and merged using FLASH (version 1.2.7) software with the following criteria: (i) the reads were truncated at any site receiving an average quality score <20 over a 50 bp sliding window; (ii) gene sequences that overlap being longer than 10 bp were merged according to their overlap with mismatch no more than 2 bp; (iii) sequences of each sample were separated according to barcodes and primers, and reads containing ambiguous bases were removed. Then, operational taxonomic units (OTUs) were clustered with 97% similarity cutoff using UPARSE (version 7.1) that can filter chimeras and cluster OTUs simultaneously. The taxonomy of each 16S rRNA gene sequence was analyzed by RDP Classifier algorithm 2.12 against the Silva (SSU123) 16S rRNA database using confidence threshold of 80%. Raw data have been deposited in the sequence read archive (SRA) at the NCBI under the accession numbers SRP145054.

### Bacterial Quantitation in Feces

In addition to illumina sequencing of 16S rRNA gene (V3-V4 regions), the total fecal bacterial load was quantified by quantitative PCR (qPCR). The 16S rRNA primes were used for measurement of total bacteria: 5′-GCAGG CCTAACACATGCAAGTC-3′ and 5′-CTGCTGCCTCCCGTA GGAGT-3′. Primes were commercially synthesized by Invitrogen (Shanghai, China). The protocol was performed using one-step SYBR Premix Ex Taq^TM^ kit (Takara, Japan). Each reaction consisted of 25 μL mixture: 12.5 μL SRBR Premix Ex Taq^TM^, 1 μL each of 10 μmol/L forward and reverse primers, 5 μL diluted template DNA at concentration of 30 ng/μL, and 5.5 μL RNase free H_2_O. The amplification was performed on a real-time PCR (ABI 7900, United States) with cycling conditions as follows: denaturation at 95°C for 15 min, followed by 40 cycles with 30 s at annealing temperature, and extension at 72°C for 30 s. Standard curve were constructed by PCR product of the 16S rRNA gene of *E.coli* as described previously (Castillo et al., 2006). Transcript copy numbers were calculated by standard curves and total bacteria were expressed as log gene copies/g in fecal sample.

### Quantification of Fermentation Products

Fecal samples stored at -80°C were used to quantify fermentation products, including lactate, acetate, propionate, isobutyrate, butyrate, isovalerate, and valerate. Samples were thawed on wet ice, then 8 mL deionized water was added to fecal sample (0.5 g), and the mixture was thoroughly homogenized by vortexing for 1 min. After heating in an ultrasonic bath for 30 min, the samples were centrifuged at 13,000 × *g* for 5 min. The supernatant was diluted 50 times and filtered through a 0.22-μm filter. Extracted sample solution (25 μL) was analyzed by a high-performance ion chromatography of ICS-3000 (Dionex, United States) as described by Yu et al. (2017).

### Statistical Analysis

Data on growth performance, nutrient digestibility, plasma indices of gut hormones and immunity, and fermentation products were analyzed using student’s *t*-test of SPSS 19.0 (Chicago, IL, United States), and the results were presented as mean values ± SEM. Microbiota diversity metrics were performed from normalized OTU reads using R software (version 3.2.2). The relative abundances of bacterial members at the level of phyla and genera were analyzed by wilcoxon rank-sum test for two-group comparisons. Differences were considered significant at *P* < 0.05.

## Results

### Growth Performance and Nutrient Digestibility

The effect of dietary CB on growth performance was evaluated (**Table 1**). There were no differences in ADFI, ADG, and FCR between CON and CB groups on days 14, 28, or throughout the trial (*P* > 0.05). To verify the status of macronutrient absorption, the apparent total tract digestibility of nutrients of GE, DM, OM, CP, EE, and TDF were measured when CB was included in diet (**Table 2**). No significant different on digestibility of the measured nutrients was observed (*P* > 0.05).

**Table 1:** The effect of dietary corn bran on growth performance in weaned pigs.

	Dietary treatment (1–14 days)			Dietary treatment (14–28 days)			Dietary treatment (1–28 days)		
			
Items	CON	CB	*P*-value	CON	CB	*P*-value	CON	CB	*P*-value
ADFI, g	478 ± 45.6	474 ± 43.9	0.948	683 ± 43.5	662 ± 26.6	0.680	573 ± 43.7	560 ± 35.4	0.832
ADG, g	289 ± 29.2	316 ± 30.6	0.541	419 ± 14.8	418 ± 8.45	0.973	349 ± 20.5	366 ± 14.5	0.513
FCR	1.66 ± 0.07	1.51 ± 0.07	0.135	1.63 ± 0.06	1.58 ± 0.07	0.640	1.64 ± 0.05	1.52 ± 0.05	0.153


*Thirty weaned pigs per treatment (six pens of five piglets) were analyzed for growth performance on days 14, 28, and the whole trial. The results were presented as mean values ± SEMs. Data were analyzed by student’s t-test. CON, control group; CB, corn bran group; ADFI, average daily feed intake; ADG, average daily gain; FCR, feed conversion ratio.*

**Table 2:** The effect of dietary corn bran on nutrient digestibility in weaned pigs.

	Dietary treatment		
		
Items, %	CON	CB	*P*-value
GE	82.0 ± 1.12	80.1 ± 0.41	0.145
DM	82.3 ± 0.91	81.0 ± 0.40	0.095
OM	84.7 ± 0.92	82.7 ± 0.37	0.074
CP	80.0 ± 2.00	77.6 ± 0.76	0.294
EE	72.0 ± 1.42	72.4 ± 1.31	0.854
TDF	60.4 ± 2.12	60.8 ± 1.62	0.862


*Fecal samples (*n* = 6 per treatment) were analyzed for nutrient digestibility on the trial of day 28. The results were presented as mean values ± SEMs. Data were analyzed by student’s t-test. CON, control group; CB, corn bran group; GE, gross energy; DM, dry matter; OM, organic matter; CP, crude protein; EE, ether extract; TDF, total dietary fiber.*

### Indices in Blood Plasma

Indices related to gut hormones and immunity were evaluated in blood plasma (**Table 3**). The endogenous gut hormones of GLP1, PYY, and 5-HT play important roles in regulation of satiety, glucose homeostasis, and gut motility. In the present study, the systemically concentrations of GLP1, PYY, and 5-HT had no differences due to dietary treatments. In addition, non-digestible but fermentable CB was expected to reduce the inflammatory response in the post-weaning piglets. Result showed that the contents of IgA, IgG, IgM, TNF-α, IL-1β, and IL-6 were not affected by the long-term intervention of CB (*P* > 0.05), however, a significant increase of IL-10 production was observed in piglets provided with CB inclusion (*P* < 0.05).

**Table 3:** The effect of dietary corn bran on indices of gut hormones and immunity in blood plasma of weaned pigs.

	Dietary treatment		
		
Items	CON	CB	*P*-value
**Gut hormones**			
GLP1, ng/L	436 ± 17.4	421 ± 8.26	0.472
PYY, ng/mL	1.58 ± 0.16	1.53 ± 0.19	0.836
5-HT, ng/mL	518 ± 55.8	433 ± 33.2	0.227
**Immune indices**			
IgA, g/L	0.68 ± 0.03	0.62 ± 0.01	0.644
IgG, g/L	21.0 ± 0.50	20.9 ± 0.19	0.816
IgM, g/L	2.36 ± 0.02	2.41 ± 0.02	0.144
TNF-α, pg/mL	69.4 ± 3.18	66.3 ± 5.66	0.644
IL-1β, pg/mL	37.3 ± 3.38	35.4 ± 2.86	0.679
IL-6, pg/mL	158 ± 12.1	155 ± 10.2	0.845
IL-10, pg/mL	22.2 ± 1.97^b^	32.5 ± 1.80^a^	0.003


*Smaples of blood plasma (*n* = 6 per treatment) were analyzed for the concentrations of gut hormones and immune indices on the trial of day 28. The results were presented as mean values ± SEMs. Data were analyzed by student’s t-test. Statistically significant differences are annotated with different letters *P* < 0.05. CON, control group; CB, corn bran group; GLP1, glucagon-like peptide 1; PYY, peptide YY; 5-HT, 5-hydroxytryptamine; IgA, immunoglobulin A; IgG, immunoglobulin G; IgM, immunoglobulin M; TNF-α, tumor necrosis factor α; IL-1β, interleukin 1β; IL-6, interleukin 6; IL-10, interleukin 10.*

### The Richness and Biodiversity of Gut Bacterial Communities

**FIGURE 1:**
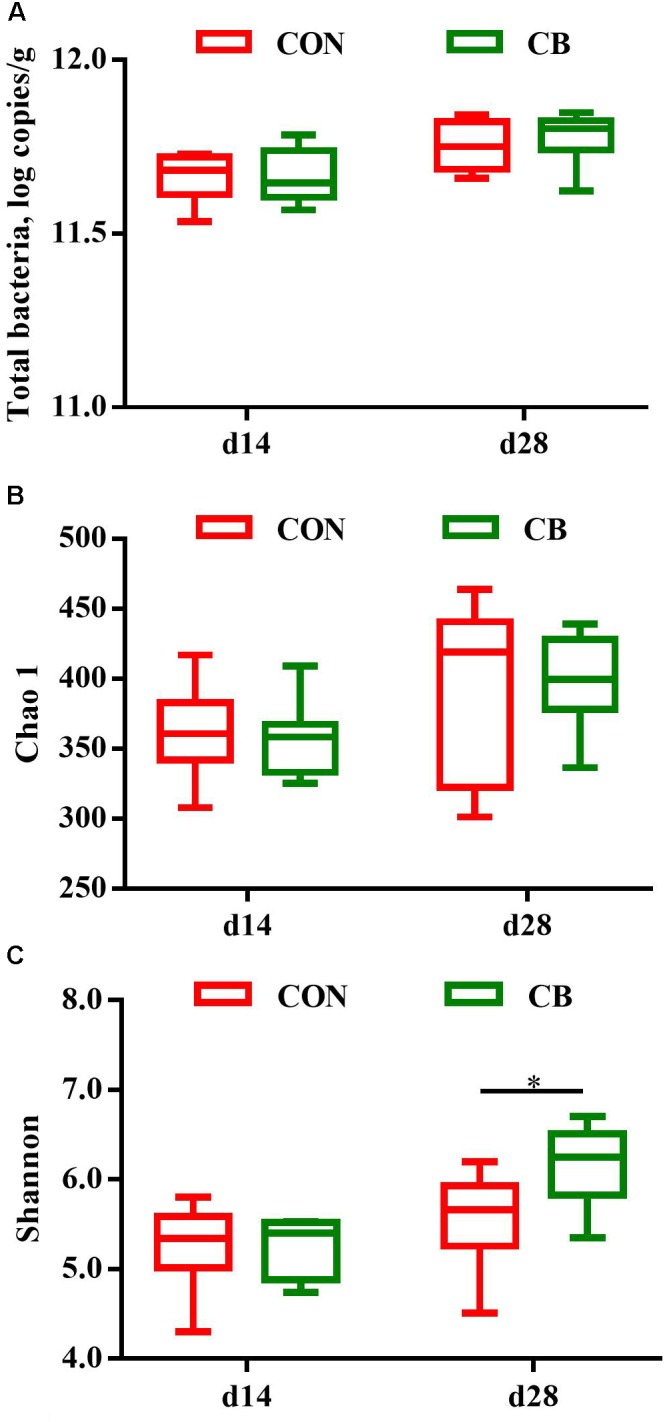
Effects of dietary corn bran on the richness and diversity of microbial communities in weaned pigs. **(A)** Total bacterial load on days 14 and 28. **(B)** Chao 1 index of bacterial community on days 14 and 28. **(C)** Shannon index of bacterial community on days 14 and 28. Gut microbiota composition in feces (*n* = 6 per treatment) were determined by 16S rRNA amplicon sequencing on the trial of days 14 and 28. The results were analyzed by wilcoxon rank-sum test and presented as mean values, and one asterisk means *P* < 0.05. CON, control group; CB, corn bran group.

Total bacteria of fecal samples were quantified in two experimental groups by qPCR. The transcript copy number of total bacteria was not significantly different (*P* > 0.05) between the experiment groups on days 14 or 28 (**Figure 1A**). Operational taxonomic units were clustered for bacterial composition on the basis of 16S rRNA gene sequencing, and the indices of chao 1 and shannon are primary criteria for assessing bacterial richness and diversity. Chao 1 index of α-diversity was not influenced by CB treatment on days 14 or 28 (*P* > 0.05, **Figure 1B**). Shannon index was not significant between the experimental groups on day 14, but CB treatment had a higher shannon index compared with CON group on day 28 (*P* < 0.05, **Figure 1C**), suggesting more diversity of bacterial communities in piglets treated with CB incorporation.

**FIGURE 2:**
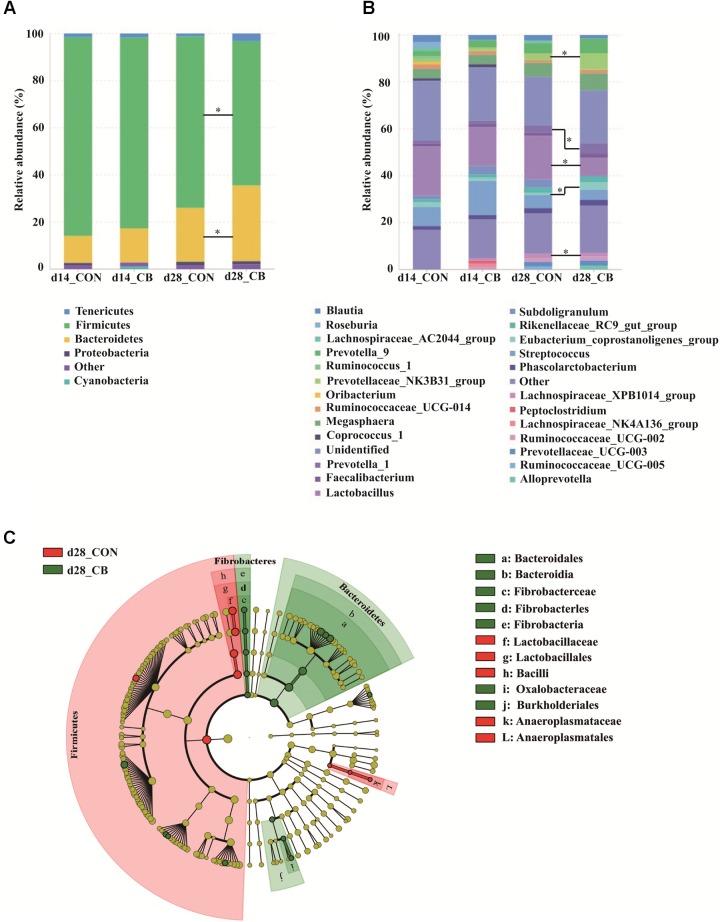
Effects of dietary corn bran on microbial community structure in weaned pigs. **(A)** Microbial community barplot at the phylum level on days 14 and 28. **(B)** Microbial community barplot at the genus level on days 14 and 28. **(C)** Cladogram of LEfSe demonstrates taxonomic profiling for the distinct bacteria with significant higher abundances on day 28. The circle from inner to outer represents the different levels of bacteria members ranged from phylum to genus. Gut microbiota composition in feces (*n* = 6 per treatment) were determined by 16S rRNA amplicon sequencing on the trial of days 14 and 28. The results were analyzed by wilcoxon rank-sum test and presented as mean relative abundance, and one asterisk means *p* < 0.05. CON, control group; CB, corn bran group; LEfSe, linear discriminant analysis effect size.

*Firmicutes* and *Bacteroidetes* were the two main phyla of bacteria in the fecal samples of weaned pigs, and their total relative abundance were approximate 95% in both experimental groups on day 14 and in CON group on day 28, except a reduced abundance of 93.42% in CB group on day 28 (**Figure 2A**). On day 14, the relative abundance of bacteria at the level of phylum (**Supplementary Table 3**) was not affected by CB treatment in weaned pigs (*P* > 0.05). However, on day 28, compared with CON group, the population of the *Firmicutes* was decreased in CB treatment (*P* < 0.05), while the relative abundance of the *Bacteroidetes* was increased (*P* < 0.05). *Clostridiales* and *Lactobacillales* were the two main orders within the *Firmicutes* phylum, whereas the *Bacteroidales* order was dominated within the *Bacteroidetes* phylum (**Supplementary Tables 4, 5**).

Moreover, gut microbiota composition in piglets treated with CON and CB supplementation were analyzed at the genus level on days 14 and 28 (**Figure 2B**). On day 14, there were no differences in the two experimental groups (*P* > 0.05). On day 28, compared with the CON group, the relative abundances of *Lactobacillus and Lachnospiraceae_AC2044_group* were decreased (*P* < 0.05), whereas the population of *Eubacterium_coprostanoligenes_group, Prevotellaceae_NK3B31_group, Prevotella_1*, and *Fibrobacter* were increased in CB treatment (*P* < 0.05, **Supplementary Table 5**). In addition, the cladogram of linear discriminant analysis effect size (LEfSe) (**Figure 2C**) generally showed that the bacterial members with significant higher abundance mainly were belonged to the *Firmicutes* phylum in CON group, whereas the bacterial taxa with significant greater population were dominated in the *Bacteroidetes* and *Fibrobacteres* phylum in CB treatment.

### Concentrations of Fermentation Products

DF is mainly fermented into SCFAs by the gut microbiota. Consequently, SCFAs and other fermentation products were quantified via high-performance ion chromatography, including lactate, acetate, propionate, isobutyrate, butyrate, isovalerate, and valerate (**Table 4**). Lactate was not detectable in the majority of fecal samples in this study, and the data was not included in table. Other detected fermentation products were not influenced by the CB inclusion on days 14 or 28 (*P* > 0.05).

**Table 4:** The effect of dietary corn bran on fermentation products in weaned pigs.

	Dietary treatment (day 14)			Dietary treatment (day 28)		
		
Item, mg/g	CON	CB	*P*-value	CON	CB	*P*-value
Acetate	3.57 ± 0.17	3.14 ± 0.20	0.137	3.53 ± 0.22	3.63 ± 0.16	0.716
Propionate	2.38 ± 0.11	2.03 ± 0.16	0.098	2.22 ± 0.09	2.46 ± 0.11	0.119
Isobutyrate	0.15 ± 0.01	0.16 ± 0.02	0.861	0.20 ± 0.02	0.21 ± 0.02	0.653
Butyrate	1.29 ± 0.11	1.08 ± 0.14	0.272	1.34 ± 0.06	1.50 ± 0.11	0.291
Isovalerate	0.14 ± 0.01	0.13 ± 0.03	0.592	0.20 ± 0.02	0.20 ± 0.02	0.843
Valerate	0.38 ± 0.06	0.28 ± 0.05	0.225	0.46 ± 0.05	0.57 ± 0.70	0.246
Total VFAs	7.92 ± 0.38	6.82 ± 0.47	0.100	7.91 ± 0.33	8.63 ± 0.28	0.123


*Fecal samples (*n* = 6 per treatment) were analyzed for fermentation products on the trial of days 14 and 28. The results were presented as mean values ± SEMs. Data were analyzed by student’s t-test. CON, control group; CB, corn barn group; VFAs, volatile fatty acids.*

## Discussion

Corn bran is an important co-product from agricultural crops, which has been widely used as an ingredient in animal production. This insoluble fiber in cereal was proposed to mitigate intestinal disorders and promote the establishment of a healthy and stable microbial ecosystem in weaning pigs (Molist et al., 2014). Weaning is critical period in pig’s life, and piglets suffer various stresses, such as a forced separation from the sow, a new living environment and a new diet, these situations lead to a transitory period of anorexia (Lalles et al., 2007). Stress and reduced feed intake normally result in gut inflammation, which affect intestinal microbial balance, the activities of enzyme and immunity in the small intestine, increasing risk of diarrhea (Pie et al., 2004). Previous study indicated more than 2 weeks is required for piglets to adapt to the weaning diet (Gresse et al., 2017). Therefore, the effects of dietary CB on animal growth and gut microbiota composition were evaluated in a long-term intervention, and time points on days 0, 14, and 28 post-weaning were focused in this study.

The effects of DF on growth performance are not consistent in pigs. Many studies have shown that high levels of DF increased daily feed intake (Gerritsen et al., 2012; Yu et al., 2016), while others reported that it has no impact on growth performance but reduced digestibility of nutrients and energy (Chen et al., 2014; Jaworski et al., 2017). In the present study, ADFI, ADG, and FCR were not influenced in the whole feeding trial, suggesting no negative effects of 5% CB inclusion on animal growth. It is consistent with the previous study (Zhao J. et al., 2018). Indeed, different DF could exert various physiological effects on the microbial composition and host cells, and the diverse effects on animal growth and nutrient digestibility were closely associated with intrinsic physicochemical characteristics of fibers. Main sources of soluble and readily fermentable fiber are from vegetables and fruits, whereas cereal fibers are mostly insoluble and low fermentable (Weickert et al., 2006). Hydration of DF is key factor to change physicochemical properties of digesta and impact macronutrient absorption. In piglets, soluble DF can increase viscosity of digesta and slow-down the gut transit time during the first days after weaning (Hopwood et al., 2004). Conversisely, insoluble DF can reduce the digesta transit time resulting in less nutrient absorption in the small intestine (Gerritsen et al., 2012). In this study, the digestibility of GE, DM, OM, CP, EE, and TDF were not significantly decreased in CB treatment, this was consistent with previous studies that suggested the level of fiber incorporation should be less than 5% to prevent reduction in nutrient digestibility of piglets (Chen et al., 2013; Han et al., 2017). Both soluble and insoluble polysaccharides can be digested by bacteria in the different segments of the large intestine according to their fermentable capacities and glycan structures, and consequently in various productions of SCFAs. Propionate and butyrate can activate GPR43 on enteroendocrine L-cells to stimulate the secretion of gut hormones, such as PYY and GLP1, which play important roles in appetite regulation via the gut–brain axis (Chen J. et al., 2015). High-fiber consumption increased the production of PYY and GLP followed by increasing satiety and inhibition of energy intake (Chambers et al., 2015). Moreover, gut contains much of the body’s 5-hydroxytryptamine (5-HT), and gut-derived 5-HT regulates gastrointestinal motility (Gershon and Tack, 2007; Zhang et al., 2018) and secretory reflexes in the host (Yano et al., 2015). In this study, the levels of GLP1, PYY, and 5-HT were not influenced by a long-term intervention of CB inclusion, and the results were coincided with effects on animal growth.

The roles of gut microbiota on health and performance in pigs, such as nutrient metabolism, stimulation of immune response, and protection from pathogens, have becoming increasingly apparent (Lalles et al., 2007; Thompson et al., 2008; Gresse et al., 2017). Diets rich in fiber can improve the diversity of gut microbiota and stimulate growth of some beneficial microbes, such as *Lactobacillus* and *Bifidobacterium* (Chen H. et al., 2015). On day 14, the composition of bacterial communities of weaned pigs did not change significantly, which might be attributed to the adaption phase of immature digestive tract after weaning. On day 28, we found higher bacterial diversity in piglets fed with CB treatment in the later phase of the feeding trial, and the result suggested that CB inclusion had beneficial effects on the establishment of a healthy and stable gut microbiota in a long-term intervention. In humans, low fiber in diet decreased the sorts of bacterial members and impair their intrinsic functions to breakdown less fermentable fiber, and the missing microbes caused by low DF consumption could not be fully restored despite switching to a high-fiber diet (Sonnenburg et al., 2016). Therefore, early bacteria colonization and succession in the gut is critical in the establishment of specific bacterial population and sharping of the host phenotype (Mach et al., 2015; Ma et al., 2018). The *Firmicutes* and *Bacteroidetes* phyla were dominant numerically in pigs with the relative abundance higher than approximately 95%. This was consistent with previous findings in weaned and finishing pigs (Schokker et al., 2014; Heinritz et al., 2016). On day 28, the total relative of *Firmicutes* and *Bacteroidetes* were decreased to approximately 93% in pigs fed diet included 5% CB, this should be associated with more bacteria members in the digestive tract, such as *Fibrobacteres*. The population of *Firmicutes* phylum was decreased, while the percentage of *Bacteroidetes* phylum was increased by dietary CB administration. This result was in agreement with a previous study in which fiber-rich diets increased the abundance of *Bacteroidetes*, especially the genera of *Xylanbacte*r (De Filippo et al., 2010). The increased population of *Bacteroidetes* might be associated with CB composition. Cellulose, hemicellulose, lignin, waxes, pectin, and proteins in plant form a complex network that resists attack from bacteria and fungi (Flint and Bayer, 2008). Gram-negative bacteria of *Bacteroidetes* have their unique polysaccharide utilization loci (PULs), and these gene clusters can generate numerous carbohydrate-enzymes, including polysaccharide lyase, polysaccharide hydrolase, and glycoside hydrolases, to cleave the linkages in the structure of glycan molecules (Sonnenburg et al., 2010).

On day 28, 5% CB inclusion showed some alterations on the abundance of bacterial communities at the genus level in weaned pigs. *Lactobacillus* is identified as a beneficial microbe to modulate gut health, and polysaccharide extracted from plants can stimulate the proliferation of *Lactobacillus* (Chen H. et al., 2015). Conversely, the population of *Lactobacillus* was significantly decreased in feces of CB group in the present study. Indeed, the relative abundance of *Lactobacillus* is reduced as age increases in young animals (Chen et al., 2017), which might be associated with the development of the immune system. Thereby, the reduced population of *Lactobacillus* might be due to the proliferation of other bacteria related with polysaccharide metabolism induced by inclusion of CB in diet. *Eubacterium coprostanoligenes* is a cholesterol-reducing bacteria and its cholesterol-lowering potential has been explored (Gerard, 2013). *E. coprostanoligenes* could be transferred from mothers to their piglet’s intestine via feces or suckling (Mach et al., 2015). In our study, an increased abundance of *E._coprostanoligenes_group* might suggest its regulatory role in lipid metabolism. The *Prevotellaceae* is a dominant family within the *Bacteroidetes* phylum producing various enzymes, such as xylanases, mannanases, β-glucanases, that can hydrolyze the polysaccharides of arabinoxylan in the cereal cell wall (Flint and Bayer, 2008). The major component of CB in this study is arabinoxylan, and the relative abundance of *Prevotella_1* and *prevotellaceae_NK3B31_group* were significantly higher in CB dietary treatment on day 28 than CON group. Notably, the relative abundance of *Fibrobacter* genus was significantly greater in CB group compared with CON group, albeit in a small proportion. *Fibrobacter* belongs to the poorly defined *Fibrobacteres* phylum, which is characterized by high cellulolytic activity and capable of degrading complex plant fiber (Qi et al., 2005). It was reported that increased abundance of *Fibrobacter* resulted in high concentration of SCFAs in rumen of dairy cows (Deng et al., 2017). Meanwhile, the population of *Fibrobacter* was increased in finishing pigs fed a diet containing 10% CB in our previous study (Liu et al., 2017). *Fibrobacte*r might be a distinct bacteria response to CB incorporation. The interplay between *Fibrobacter* and CB metabolism in humans and monogastric animals needs extra attention. Besides of the distinct bacteria with higher abundances, a reduction on the population of *Lachnospiraceae_AC2044_group* was observed in piglet supplied CB treatment, the reason for this alteration might be associated with the increased microbiota members of *Prevotellaceae* and other fibrolytic bacteria.

Die-induced modulations on gut microbiota composition could impact on fermentative activity of substrates in the gut, for instance, high DF increased SCFAs production, particularly the butyrate concentration (Chambers et al., 2015; Liu et al., 2018). In the preset study, there was no significant responses to CB on fermentation products in feces on days 14 or 28, although a few alterations in the microbial communities that were discussed above. It is uncertain that SCFAs concentrations in digesta are positively associated with that in feces. Weaning induces intestinal dysbiosis accompanied by increased pro-inflammatory cytokines in piglets, such as IL-1β, IL-6, and TNF-α (Pie et al., 2004). Controlling the production of pro-inflammatory cytokines might have potential benefits in suppressing gut mucosal inflammation. Microbial activities derived from DF can bind to innate immune cells followed by enhanced antibody response (Kim et al., 2016; Wang et al., 2016) and reduction of inflammatory makers (Daien et al., 2017). In our study, the contents of IgA, IgG, IgM, IL-1β, IL-6, and TNF-α were not affected by CB treatment. IL-10 is anti-inflammatory cytokines producing by T cells via butyrate-stimulated signaling of GPR109a (Singh et al., 2014). Notably, the production of IL-10 was increased in CB group suggesting that CB could promote anti-inflammatory response to some extent in weaned pigs. In particular, IL-10 is critical to maintain the goblet cell production of mucins (Hasnain et al., 2013), and the mutations in IL-10 receptors resulted in inflammatory bowel diseases (Begue et al., 2011). Thereby, an increased production of IL-10 hints that CB might involve in activities on the first protective barrier of mucus. Whether CB impacts mucin secretion in an IL-10 dependent manner should be highly emphasized in further study.

## Conclusion

In conclusion, weaner diet containing 5% CB did not impact growth performance, nutrient digestibility, concentrations of gut hormones, and fermentation products in feces, but promoted the anti-inflammatory response in weaned pigs. The diversity of gut microbiota was increased via a long-term CB intervention. In particular, *Eubacterium corprostanoligenes, Pevotella*, and *Fibrobacter* related to polysaccharide fermentation were distinct bacteria with higher abundances. These changes should facilitate the adaptation of the digestive system of piglets in the subsequent growing phases. The underlying mechanisms on how the main components of CB affect bacteria related to the metabolism of polysaccharide and lipid will be focused on in further investigation.

## Author Contributions

XM and WL designed the experiments. PL, JZhao, and PG performed the experiments. LL and CW analyzed the data. PL wrote the manuscript. WW, LJ, YZ, XW, JZhang, and CX gave a critical reading and modification. XM resourced the project. All authors read and approved the final manuscript.

## Conflict of Interest Statement

The authors declare that the research was conducted in the absence of any commercial or financial relationships that could be construed as a potential conflict of interest.
