# Supplementary material for: Dietary Corn Bran Altered the Diversity of Microbial Communities and Cytokine Production in Weaned Pigs
Source: Front Microbiol. 2018 Sep 4;9:2090. doi: 10.3389/fmicb.2018.02090 (PMC6131307; doi:10.3389/fmicb.2018.02090)
Supplement: Supplementary file 1 [file Table_1.doc]

**Supplemental Table 1. The composition of experimental diets and nutritional values (as-fed basis)^1^**

|  | Dietary treatment | | | |
| --- | --- | --- | --- | --- |
| Items | CON | | | CB |
| Ingredients, %  Corn | 55.02 | | | 50.12 |
| Dehulled soybean meal | 15.70 | | | 15.30 |
| Extruded full-fat soybean | 5.00 | | | 5.00 |
| Corn barn | - | | | 5.00 |
| Soy protein concentrate | 4.00 | | | 4.00 |
| Fish meal | 4.00 | | | 4.00 |
| Whey powder | 8.00 | | | 8.00 |
| Sucrose | 3.00 | | | 3.00 |
| Zinc oxide | 0.28 | | | 0.28 |
| Soybean oil | 1.30 | | | 1.50 |
| Dicalcium phosphate | 1.20 | | | 1.20 |
| Limestone | 0.50 | | | 0.50 |
| Salt | 0.30 | | | 0.30 |
| Lysine | 0.30 | | | 0.35 |
| Methionine | 0.20 | | | 0.25 |
| Threonine | 0.15 | | | 0.18 |
| Tryptophan | 0.10 | | | 0.10 |
| Valine | 0.20 | | | 0.25 |
| Chromic oxide | 0.25 | | | 0.25 |
| Vitamin-mineral premix^2^ | 0.50 | | | 0.50 |
| Total | 100 | | | 100 |
| Nutritional level | |  | | |
| Digestive energy，MJ/kg | 14.50 | | 14.50 | |
| Crude protein | 18.50 | | 18.50 | |
| Total dietary fiber | 18.40 | | 22.10 | |
| Calcium | 0.80 | | 0.80 | |
| Phosphorus | 0.60 | | 0.60 | |
| SID Lysine | 1.30 | | 1.30 | |
| SID Methionine + Cysteine | 0.80 | | 0.80 | |
| SID Threonine | 0.90 | | 0.90 | |
| SID Tryptophan | 0.30 | | 0.30 | |

^1^CON, control group; CB, corn bran group; SID, standardized ileal digestible.

^2^Premix provided the following per kg of complete diet for growing pigs: vitamin A, 5,512 IU; vitamin D_3_, 2,200 IU; vitamin E, 30 IU; vitamin K_3_, 2.2 mg; vitamin B12, 27.6 μg; riboflavin, 4.0 mg; pantothenic acid, 14.0 mg; niacin, 30.0 mg; choline chloride, 400.0 mg; folacin, 0.7 mg; thiamine 1.5 mg; pyridoxine 3.0 mg; biotin, 44.0 ug; Mn (MnO), 40.0 mg; Fe (FeSO_4_•H_2_O), 75.0 mg; Zn (ZnO), 75.0 mg; Cu (CuSO_4_•5H_2_O), 100.0 mg; I (KI), 0.3 mg; Se (Na_2_SeO_3_), 0.3 mg.
