# Supplementary material for: Dietary Corn Bran Altered the Diversity of Microbial Communities and Cytokine Production in Weaned Pigs
Source: Front Microbiol. 2018 Sep 4;9:2090. doi: 10.3389/fmicb.2018.02090 (PMC6131307; doi:10.3389/fmicb.2018.02090)
Supplement: Supplementary file 2 [file Table_2.doc]

**Supplemental Table 2. Fiber composition of corn bran (dry matter basis)^1^**

| Items, g/kg | CB |
| --- | --- |
| Starch | 243.2 |
| Organic matter | 971.7 |
| Crude protein | 164.8 |
| Cellulose | 120.1 |
| Hemicellulose | 332.7 |
| Lignin | 22.9 |
| Total dietary fiber | 601.3 |
| SDF | 66.7 |
| IDF | 534.7 |
| SDF/IDF | 12.5 |
| NSP | 535.5 |
| Rhamnose | 3.5 |
| Fructose | 2.0 |
| Ribose | 3.0 |
| Arabinose | 89.5 |
| Xylose | 148.0 |
| Mannose | 18.5 |
| Galactose | 24.5 |
| Glucose | 246.5 |

^1^CB, corn bran; SDF, soluble dietary fiber; IDF, insoluble dietary fiber; NSP, non-starch polysaccharides.
