# Supplementary material for: Dietary Corn Bran Altered the Diversity of Microbial Communities and Cytokine Production in Weaned Pigs
Source: Front Microbiol. 2018 Sep 4;9:2090. doi: 10.3389/fmicb.2018.02090 (PMC6131307; doi:10.3389/fmicb.2018.02090)
Supplement: Supplementary file 3 [file Table_3.docx]

**Supplemental Table 3. The effect of dietary corn bran on intestinal microbiota composition of weaned piglets at the phylum level^1^**

|  | Dietary treatment | |  |
| --- | --- | --- | --- |
| Taxa | CON | CB | *P-*value |
| On 14 d |  |  |  |
| *Firmicutes* | 84.59 | 81.03 | > 0.05 |
| *Bacteroidetes* | 11.21 | 14.37 | > 0.05 |
| *Tenericutes* | 1.64 | 1.76 | > 0.05 |
| *Proteobacteria* | 1.00 | 0.57 | > 0.05 |
| *Actinobacteria* | 0.78 | 0.68 | > 0.05 |
| *Cyanobacteria* | 0.19 | 1.18 | > 0.05 |
| *Spirochaetae* | 0.51 | 0.15 | > 0.05 |
| *Fibrobacteres* | 0.03 | 0.14 | > 0.05 |
| *SHA-109* | 0.01 | 0.07 | > 0.05 |
| *Euryarchaeota* | 0.01 | 0.03 | > 0.05 |
| *Saccharibacteria* | 0.02 | 0.01 | > 0.05 |
| On 28 d |  |  |  |
| *Firmicutes* | 72.93 | 61.58 | < 0.05 |
| *Bacteroidetes* | 22.75 | 31.84 | < 0.05 |
| *Tenericutes* | 1.25 | 3.31 | > 0.05 |
| *Proteobacteria* | 1.62 | 1.26 | > 0.05 |
| *Spirochaetae* | 0.70 | 0.71 | > 0.05 |
| *Actinobacteria* | 0.52 | 0.76 | > 0.05 |
| *Cyanobacteria* | 0.13 | 0.23 | > 0.05 |
| *Fibrobacteres* | 0.05 | 0.24 | < 0.05 |
| *Saccharibacteria* | 0.02 | 0.04 | > 0.05 |
| *SHA-109* | 0.02 | 0.01 | > 0.05 |
| *Lentisphaerae* | 0.00 | 0.02 | > 0.05 |
| *Euryarchaeota* | 0.01 | 0.01 | > 0.05 |

^1^ Gut microbiota composition in feces (n = 6 per treatment) were determined by 16S rRNA amplicon sequencing on the trial of d 14 and d 28. The results were analyzed by wilcoxon rank-sum test and presented as mean relative abundance of order bacteria. CON, control group; CB, corn bran group.
