# Supplementary material for: Dietary Corn Bran Altered the Diversity of Microbial Communities and Cytokine Production in Weaned Pigs
Source: Front Microbiol. 2018 Sep 4;9:2090. doi: 10.3389/fmicb.2018.02090 (PMC6131307; doi:10.3389/fmicb.2018.02090)
Supplement: Supplementary file 4 [file Table_4.docx]

**Supplemental Table 4. The effect of dietary corn bran on intestinal microbiota composition of weaned piglets at the genus level on d 14^1^**

| Taxa | | | | | Dietary treatment | | |
| --- | --- | --- | --- | --- | --- | --- | --- |
| Phylum | Class | Order | Family | Genus | CON | CB | *P*-value |
| *Firmicutes* | *Bacilli* | *Lactobacillales* | *Lactobacillaceae* | *Lactobacillus* | 20.59 | 16.60 | > 0.05 |
|  |  |  | *Streptococcaceae* | *Streptococcus* | 8.00 | 14.51 | > 0.05 |
|  | *Clostridia* | *Clostridiales* | *Lachnospiraceae* | *Pseudobutyrivibrio* | 15.09 | 7.56 | > 0.05 |
|  |  |  |  | *Blautia* | 3.11 | 2.26 | > 0.05 |
|  |  |  |  | *Coprococcus_1* | 1.10 | 1.36 | > 0.05 |
|  |  |  |  | *Lachnospiraceae_UCG-005* | 1.70 | 0.29 | > 0.05 |
|  |  |  |  | *Oribacterium* | 1.07 | 0.83 | > 0.05 |
|  |  |  |  | *Lachnospiraceae_AC2044_group* | 1.57 | 0.18 | > 0.05 |
|  |  |  |  | *Lachnospira* | 0.84 | 0.90 | > 0.05 |
|  |  |  |  | *Lachnospiraceae_NK4A136_group* | 0.46 | 1.18 | > 0.05 |
|  |  |  |  | *Lachnospiraceae_XPB1014_group* | 0.24 | 1.18 | > 0.05 |
|  |  |  | *Ruminococcaceae* | *Subdoligranulum* | 1.69 | 3.70 | > 0.05 |
|  |  |  |  | *Ruminococcaceae_UCG-014* | 2.04 | 1.69 | > 0.05 |
|  |  |  |  | *Eubacterium_coprostanoligenes_group* | 2.07 | 1.37 | > 0.05 |
|  |  |  |  | *Faecalibacterium* | 1.46 | 1.18 | > 0.05 |
|  |  |  |  | *Ruminococcus_1* | 1.12 | 1.02 | > 0.05 |
|  |  |  |  | *Ruminococcaceae_UCG-002* | 0.83 | 1.15 | > 0.05 |
|  |  |  |  | *Ruminococcaceae_UCG-008* | 0.98 | 0.91 | > 0.05 |
|  |  |  |  | *Ruminococcaceae_UCG-005* | 0.96 | 0.72 | > 0.05 |
|  | *Negativicutes* | *Selenomonadales* | *Acidaminococcaceae* | *Phascolarctobacterium* | 1.60 | 1.77 | > 0.05 |
|  |  |  | *Veillonellaceae* | *Selenomonas_3* | 0.64 | 2.31 | > 0.05 |
| *Bacteroidetes* | *Bacteroidia* | *Bacteroidales* | *Prevotellaceae* | *Prevotella_9* | 2.25 | 3.24 | > 0.05 |
|  |  |  |  | *Prevotellaceae_NK3B31_group* | 1.33 | 1.37 | > 0.05 |
|  |  |  |  | *Prevotella_1* | 1.17 | 1.45 | > 0.05 |
|  |  |  | *Rikenellaceae* | *Rikenellaceae_RC9_gut_group* | 1.13 | 1.25 | > 0.05 |
| *Fibrobacteres* | *Fibrobacteria* | *Fibrobacterales* | *Fibrobacteraceae* | *Fibrobacter* | 0.03 | 0.14 | > 0.05 |

^1^ Gut microbiota composition in feces (n = 6 per treatment) were determined by 16S rRNA amplicon sequencing on the trial of d 14. The results were analyzed by wilcoxon rank-sum test and presented as mean relative abundance of genus bacteria. CON, control group; CB, corn bran group.
