# Supplementary material for: Dietary Corn Bran Altered the Diversity of Microbial Communities and Cytokine Production in Weaned Pigs
Source: Front Microbiol. 2018 Sep 4;9:2090. doi: 10.3389/fmicb.2018.02090 (PMC6131307; doi:10.3389/fmicb.2018.02090)
Supplement: Supplementary file 5 [file Table_5.docx]

**Supplemental Table 5. The effect of dietary corn bran on intestinal microbiota of weaned pigs at the genus level on d 28^1^**

| Taxa | | | | | Dietary treatment | | |
| --- | --- | --- | --- | --- | --- | --- | --- |
| Phylum | Class | Order | Family | Genus | CON | CB | *P* value |
| *Firmicutes* | *Bacilli* | *Lactobacillales* | *Lactobacillaceae* | *Lactobacillus* | 18.35 | 7.86 | < 0.05 |
|  |  |  | *Streptococcaceae* | *Streptococcus* | 5.38 | 4.36 | > 0.05 |
|  | *Negativicutes* | *Selenomonadales* | *Veillonellaceae* | *Megasphaera* | 5.67 | 6.90 | > 0.05 |
|  |  |  |  | *Selenomonas_3* | 2.21 | 1.99 | > 0.05 |
|  |  |  | *Acidaminococcaceae* | *Phascolarctobacterium* | 2.32 | 2.43 | > 0.05 |
|  | *Clostridia* | *Clostridiales* | *Ruminococcaceae* | *Subdoligranulum* | 3.65 | 0.82 | > 0.05 |
|  |  |  |  | *Eubacterium_coprostanoligenes_group* | 1.13 | 3.19 | < 0.05 |
|  |  |  |  | *Ruminococcaceae_UCG-002* | 1.67 | 1.95 | > 0.05 |
|  |  |  |  | *Faecalibacterium* | 1.49 | 1.78 | > 0.05 |
|  |  |  |  | *Ruminococcaceae_UCG-014* | 1.26 | 1.70 | > 0.05 |
|  |  |  |  | *Ruminococcaceae_UCG-005* | 1.42 | 1.13 | > 0.05 |
|  |  |  |  | *Ruminococcus_1* | 0.67 | 1.00 | > 0.05 |
|  |  |  |  | *Ruminococcaceae_NK4A214_group* | 0.64 | 0.86 | > 0.05 |
|  |  |  | *Lachnospiraceae* | *Blautia* | 2.33 | 1.52 | > 0.05 |
|  |  |  |  | *Lachnospiraceae_XPB1014_group* | 1.98 | 1.35 | > 0.05 |
|  |  |  |  | *Lachnospira* | 0.54 | 0.86 | > 0.05 |
|  |  |  |  | *Lachnospiraceae_AC2044_group* | 1.27 | 0.01 | < 0.05 |
|  |  |  | *Christensenellaceae* | *Christensenellaceae_R-7_group* | 0.43 | 0.96 | > 0.05 |
| *Bacteroidetes* | *Bacteroidia* | *Bacteroidales* | *Prevotellaceae* | *Prevotella_9* | 4.24 | 6.18 | > 0.05 |
|  |  |  |  | *Prevotellaceae_NK3B31_group* | 2.83 | 6.71 | < 0.05 |
|  |  |  |  | *Prevotella_1* | 2.44 | 3.86 | < 0.05 |
|  |  |  |  | *Rikenellaceae_RC9_gut_group* | 2.06 | 2.40 | > 0.05 |
|  |  |  |  | *Prevotellaceae_UCG-003* | 1.89 | 2.19 | > 0.05 |
|  |  |  |  | *Alloprevotella* | 0.89 | 1.56 | > 0.05 |
| *Fibrobacteres* | *Fibrobacteria* | *Fibrobacterales* | *Fibrobacteraceae* | *Fibrobacter* | 0.05 | 0.24 | < 0.05 |

^1^ Gut microbiota composition in feces (n = 6 per treatment) were determined by 16S rRNA amplicon sequencing on the trial of d 28. The results were analyzed by wilcoxon rank-sum test and presented as mean relative abundance of genus bacteria. CON, control group; CB, corn bran group.
